# Expression of serum miR-20a-5p, let-7a, and miR-320a and their correlations with pepsinogen in atrophic gastritis and gastric cancer: a case–control study

**DOI:** 10.1186/1472-6890-13-11

**Published:** 2013-03-22

**Authors:** Qian Xu, Qi-Guan Dong, Li-ping Sun, Cai-yun He, Yuan Yuan

**Affiliations:** 1 Tumor Etiology and Screening Department of Cancer Institute and General Surgery, the First Affiliated Hospital of China Medical University, the Key Laboratory of Tumor Etiology and Prevention in Liaoning Province, Shenyang, Liaoning Province 110001, China; 2 The Department of Medical Oncology, the General Hospital of Fushun Mining Bureau, Fushun, Liaoning Province 113008, China

**Keywords:** Serum microRNA, Biomarker, Pepsinogen, Gastric disease

## Abstract

**Background:**

The identification of serial miRNAs targeting the same functional gastric protein could provide new and effective serological biomarkers for the diagnosis of gastric cancer (GC). The aim of this study was to evaluate the potential of miR-20a-5p, let-7a and miR-320a in the diagnosis of AG or GC and the correlation of the three miRNAs with their predicted target molecules PGA, PGC and PGA/PGC ratio.

**Methods:**

The total of 291 patients included 103 controls (CON), 94 with atrophic gastritis (AG) and 94 with GC. The levels of serum miRNAs were detected by quantitative reverse transcription-polymerase chain reaction and serum pepsinogen A (PGA) and C (PGC) were determined by enzyme-linked immunosorbent assays.

**Results:**

Serum miR-320a level decreased through the controls, AG and GC groups which were the cascades of GC development, while there were no significant differences in levels of miR-20a-5p and let-7a among the controls, AG and GC groups. When stratified by gender and age, serum miR-320a expression was lower in female GC patients than in controls (*p* = 0.035), especially in female GC patients older than 60 years (*p* = 0.008). For distinguishing female GC patients aged over 60, the area under the receiver operating characteristic curve for miR-320a was 0.699, and the best cut-off point was 4.76 with a sensitivity of 65.2% and specificity of 68.2%. Concerning the correlations between the selected miR-20a-5p, let-7a, miR-320a and PGs, we found that there were positive correlations between all the three and the ratio of PGA/PGC (r = 0.408, 0.255, 0.324; *p* = <0.001, 0.009, 0.001, respectively), but there was no relationship between the expression of serum miR-20a-5p and its predicted target PGA, or between let-7a and miR-320a and their predicted target PGC. Serum miR-320a was decreased and PGC was increased in the GC group compared with the control group.

**Conclusions:**

Levels of serum miR-320a were lower in female GC patients older than 60 than in controls, which may provide a potential valuable marker for diagnosing older women with GC. The levels of serum miR-20a-5p, let-7a and miR-320a were positively correlated with PGA/PGC, which may indirectly reflect the functional status of the gastric mucosa.

## Background

It is essential to explore sensitive, specific and non-invasive tumour biomarkers that can be used for early diagnosis, estimating prognosis, and forecasting recurrence. MicroRNAs (miRNAs) are a class of highly conserved and endogenous non-coding RNAs 19–24 nucleotides long, which regulate the expression of target genes at the post-transcriptional level. Accumulating evidence indicates that miRNAs are involved in important biological processes related to proliferation, apoptosis, differentiation, metastasis, angiogenesis and immune response, while deregulation of miRNAs may be crucial in cancer initiation, progression and treatment outcome [1,2]. It has recently been shown that human serum or plasma contains large amounts of stable miRNAs, the expression profiles of which could potentially be used to identify various types of cancer, including prostate cancer, large B-cell lymphoma, ovarian cancer, liver cancer and non-small cell lung cancer [3-7].

miRNAs are part of a complex regulatory network; multiple genes can be regulated by one miRNA, but a single gene may also be subtly regulated by a group of miRNAs [2]. Few studies have investigated the association between serum miRNAs and gastric diseases [8,9], several studies focused on the plasma miRNAs expression [10-12], Liu H et al. identified miR-378 as a serum biomarker [8], and Liu R et al. selected five miRNAs (miR-1,-20a,-27a,-34 and −423-5p) as a fingerprint for GC diagnosis [9]. But no attention has been paid to targeting of the same gene by serial miRNAs. However, such studies could help to clarify the common functions of serial miRNAs targeting individual genes and thus improve our understanding of their roles in gastric cancer (GC) and precancerous diseases.

Human pepsinogens (PGs) are inactive pro-enzymes of the specific digestive enzyme pepsin, which originate from the gastric mucosa and can be classified biochemically and immunochemically into PGA and PGC [13,14]. Previous studies showed that PGC expression was negatively correlated with the degree of malignancy of the gastric mucosa and the development of gastric lesions [15]. A low PGA/PGC ratio can be used as a serological biomarker of atrophic gastritis (AG), which is known to be a precancerous condition [15,16]. Several miRNAs have been identified by two target gene-predicting software packages (MicroCosm Targets and TargetScan Human), some of which targeted PGA (miR-212, miR-132, miR-20a-5p, miR-93) or PGC (miR-662, miR-365, let-7a, miR-320a). The miRNAs closely related to GC or other malignancies were selected for further investigation in the current study. miR-20a-5p, a member of the miR-17-92 cluster, was upregulated in gastric, colorectal and pancreatic adenocarcinomas *in situ *[17-19], tissue expression of let-7a was correlated with lymph node metastasis of GC[20], and miR-320a was down-regulated in primary breast cancer and correlated with invasion and metastasis [21-23]. These three miRNAs have only previously been investigated *in situ*, and most studies have focused on their functions; no studies have investigated their potential roles as serum biomarkers for the diagnosis of GC and precancerous disease. And whether the predicted functional miRNAs (miR-20a-5p, let-7a and miR-320a) could be identified as a biomarker for the diagnosis of GC and precancerous disease? Furthermore, their relationships with their predicted target pepsinogen are unknown.

This study therefore aimed to evaluate the potential of miR-20a-5p, let-7a and miR-320a in the diagnosis of GC and the correlation of the three miRNAs and PGA, PGC and PGA/PGC ratio. We investigated the serum levels of the three selected miRNAs in GC and its precancerous condition, as well as the correlation between serum miR-20a-5p and its predicted target PGA, and between serum let-7a and miR-320a and their predicted target PGC. The results maybe identify several valuable serological markers for gastric diseases to some extent, as well as providing experimental evidence and reference to aid our further understanding of the functions of serum miRNAs in the process of carcinogenesis.

## Methods

### Subjects

A total of 291 subjects who underwent gastroscopy examinations in the first Affiliated Hospital of China Medical University from 2004 to 2011 were enrolled in this study. Information about gender, age and other factors was obtained by means of a questionnaire administered to each subject. The study was approved by the Human Ethics Review Committee of China Medical University. Written informed consent was obtained from participants in accordance with the Declaration of Helsinki and its later revision. All patients underwent endoscopic gastric mucosal biopsy, and the biopsy specimens were paraffin-embedded and stained by HE staining for histological diagnosis which was diagnosed by two experienced pathologists. Cases with minimal superficial gastritis were treated as the controls [24]. The atrophic gastritis group was defined according to histopathology. The histopathology diagnoses was included slight, moderate and severity, and only the grade of moderate and severity was enrolled in this case–control study. Ninety-four patients were histologically confirmed gastric adenocarcinoma cases; 75 could be classified according to Lauren classification, while 19 could not. And among the 75 cases [25], 22 of the 75 GC cases were the intestinal type, 45 were the diffuse type and 8 were the mixed type. The samples in gastric cancer group matched in the age and gender composition with patients with controls and with atrophic gastritis.

### Serum RNA isolation and quantitative reverse transcription-polymerase chain reaction (qRT-PCR) assay

Approximately 6 ml venous blood was collected from each subject. Serum total RNA was extracted using a method described by Liu R et al. [9] with some modifications. Serum was separated by centrifugation at 3,000 *g* for 10 min, followed by a 15 min high-speed centrifugation at 12,000 *g* to completely remove cell debris. The supernatant serum was recovered and stored at −80°C until further processing. Total RNA was extracted from 200 μl of serum by acid phenol/chloroform purification and centrifugation in isopropyl alcohol. Every sample was all in 20 μl RNA-free ddH_2_O solution and six microlitres of total RNA was reverse-transcribed to cDNA using One Step PrimeScript miRNA cDNA Synthesis Kit (TaKaRa, Dalian, China). Real-time PCR was performed using a miRcute miRNA qPCR detection kit (Tiangen, Beijing, China) on the Thermal Cycler Dice Real Time System (TaKaRa). To calculate the levels of the miRNAs, synthetic hsa-miR-20a-5p, hsa-let-7a and hsa-miR-320a at known concentrations (TaKaRa) were also reverse-transcribed and amplified. The concentration of each miRNA was then calculated according to the standard curve. All reactions, including no-template controls, were run in duplicate.

### Serum PG and *H. pylori* levels

Serum PG concentrations and *H.pylori* (HP) levels were determined by enzyme-linked immunosorbent assay (ELISA) with PGA, PGC and *H.pylori* ELISA kits (Biohit Co., Ltd., Helsinki, Finland) [16,26,27]. Samples were assayed in random order, blind to histology results. Each batch included commercial controls and blinded plasma controls to assess laboratory variation. All plasma controls were within the mean ± 2 SDs. Five percent of all samples were assayed in duplicate.

### Statistical analysis

The log_10 _of the miRNA copy number represented the levels of the miRNAs, and differences in the levels of miRNA among the groups as well as in the stratification analysis were compared by one-way analysis of variance. Receiver operating characteristic (ROC) curves and the area under the ROC curve (AUC) were used to evaluate the diagnostic effects of the miRNAs and to determine appropriate cut-off points. Medians serum PG concentrations were compared among the three groups as well as in the stratification analysis using Kruskal-Wallis H tests. Pearson’s correlation coefficient was used to estimate the correlation between miRNAs and PG concentrations. The scatter plot was represent the correlation analysis between the serum miRNA levels and PGA/PGC ratio, and between PGA and PGC levels. All statistical analyses were performed using SPSS 17.0 software (SPSS Inc. Chicago, USA). A two-sided *p* value < 0.05 was considered statistically significant.

## Results

### Demographics of the study subjects

The 291 subjects included 103 controls, 94 patients with AG and 94 with GC. As shown in Table 1, there were no significant differences in terms of gender (*p* = 0.948), age (*p* = 0.654) and *H.pylori* infection status (*p* = 0.580) among three groups.

**Table 1:** Characteristics of the study subjects

**Variables**	**Control**	**Atrophic gastritis**	**Gastric cancer**
	**n = 103**	**n = 94**	**n = 94**
Gender			
Male	65(63.1%)	61(64.9%)	59(62.8%)
Female	38(36.9%)	33(35.1%)	35(37.2%)
	*P* = 0.948		
Age(years)			
Mean	59.1 ± 10.6	60.4 ± 11.2	60.2 ± 11.1
Range	23-80	28-83	27-80
	*P* = 0.654		
*H.pylori* infection status			
Hp-	60(58.3%)	48(51.1%)	53(56.4%)
Hp+	43(41.7%)	46(48.9%)	41(43.6%)
	*P* = 0.580		

### Correlations between expression of serum miRNAs and gastric diseases

There were no significant differences in the levels of miR-20a-5p and let-7a among the controls, AG and GC groups (*p* = 0.581, 0.445, respectively), but miR-320a levels decreased gradually among the different groups (4.99 ± 0.46 vs. 4.94 ± 0.45 vs. 4.90 ± 0.44, *p* = 0.152, Table 2).

**Table 2:** Levels of serum miRNAs (mean ± SD), PG (median ± SD) between different gastric disease groups

**Variables**		**CON**	**AG**	**GC**	***P-*value**
miR-20a-5p		3.77 ± 0.74	3.84 ± 0.69	3.83 ± 0.68	0.581
	Male	3.80 ± 0.77	3.90 ± 0.73	3.88 ± 0.78	0.59
	Female	3.72 ± 0.69	3.73 ± 0.60	3.75 ± 0.47	0.855
let-7a		3.72 ± 0.34	3.77 ± 0.29	3.75 ± 0.28	0.445
	Male	3.72 ± 0.31	3.78 ± 0.32	3.77 ± 0.28	0.367
	Female	3.71 ± 0.39	3.76 ± 0.24	3.72 ± 0.28	0.93
miR-320a		4.99 ± 0.46	4.94 ± 0.45	4.90 ± 0.44	0.152
	Male	5.03 ± 0.49	5.01 ± 0.46	5.00 ± 0.42	0.76
	Female	4.93 ± 0.40	4.81 ± 0.39	4.73 ± 0.43	0.035^*^
PGA		146.3 ± 101.5	111.0 ± 85.4	130.1 ± 107.1	**0.044**^§^
PGC		19.2 ± 17.6	14.0 ± 12.2	23.1 ± 23.0	**0.003**^§^
PGA/PGC		9.52 ± 4.85	9.52 ± 5.23	7.86 ± 5.46	**0.039**^§^

P^*^:GC *vs* Control; *P*^*§*^:difference among the three groups. CON: Cases with minimal superficial gastritis were treated as the controls; AG: atrophic gastritis; GC: gastric cancer.

Stratification by gender and age showed that miR-320a expression was lower in female GC patients than in controls (*p* = 0.035), and the difference was especially significant in female GC patients older than 60 years, compared with controls (*p* = 0.008) (Table 3). The AUC of miR-320a for detecting female GC patients older than 60 was 0.699. The best cut-off point was 4.76, with a sensitivity of 65.2% (95%CI = 45.8–84.7%), and a specificity of 68.2% (95%CI = 48.7–87.6%). We also analysis our data in the *H.pylori* stratification, but there was no difference between any of the groups as shown in Table 4.

**Table 3:** Stratification analysis of serum miR-320a level between different gastric disease groups (mean ± SD)

**miRNA**			**CON(n)**	**AG(n)**	**GC(n)**	***P-*value**
miR-320a	Male	≥60	5.02 ± 0.44(27)	4.96 ± 0.43(28)	4.99 ± 0.46(26)	0.796
		<60	5.03 ± 0.53(38)	5.05 ± 0.49(33)	5.01 ± 0.39(33)	0.859
	Female	≥60	4.98 ± 0.46(22)	4.77 ± 0.39(24)	4.63 ± 0.41(23)	0.008^*^
		<60	4.88 ± 0.31(16)	4.91 ± 0.39(9)	4.92 ± 0.42(12)	0.774

P^*^:GC *vs* Control. n mean the sample number of each subgroup.

**Table 4:** **Expression levels of serum miRNAs (mean ± SD), PG (median ± SD) between different gastric disease groups when stratified by *H.pylori *status**

**Variables**		**CON**	**AG**	**GC**	***P-*value**
**Hp-**					
miR-20a-5p		3.83 ± 0.71	3.88 ± 0.59	3.85 ± 0.70	0.926
Male		3.84 ± 0.72	3.90 ± 0.59	3.89 ± 0.34	0.931
Female		3.82 ± 0.71	3.86 ± 0.60	3.75 ± 0.53	0.881
let-7a		3.74 ± 0.31	3.81 ± 0.31	3.76 ± 0.25	0.505
Male		3.73 ± 0.31	3.84 ± 0.34	3.77 ± 0.25	0.36
Female		3.76 ± 0.30	3.76 ± 0.28	3.74 ± 0.27	0.972
miR-320a		5.02 ± 0.48	4.90 ± 0.45	4.91 ± 0.47	0.311
Male		5.03 ± 0.50	4.97 ± 0.48	4.98 ± 0.44	0.813
Female		4.99 ± 0.44	4.81 ± 0.41	4.74 ± 0.50	0.216
PGA		134.99 ± 92.27	96.78 ± 76.05	141.99 ± 125.80	0.056
PGC		15.13 ± 13.67	10.37 ± 9.02	23.15 ± 25.11	**0.001**
PGA/PGC		10.66 ± 4.81	11.00 ± 6.04	9.17 ± 6.28	0.22
**Hp+**					
miR-20a-5p		3.69 ± 0.78	3.79 ± 0.79	3.81 ± 0.66	0.743
Male		3.75 ± 0.86	3.90 ± 0.31	3.86 ± 0.82	0.785
Female		3.62 ± 0.66	3.53 ± 0.55	3.74 ± 0.43	0.542
let-7a		3.68 ± 0.38	3.74 ± 0.27	3.74 ± 0.31	0.661
Male		3.70 ± 0.31	3.73 ± 0.30	3.77 ± 0.33	0.717
Female		3.66 ± 0.48	3.75 ± 0.18	3.70 ± 0.28	0.795
miR-320a		4.95 ± 0.44	4.98 ± 0.45	4.89 ± 0.40	0.634
Male		5.01 ± 0.49	5.05 ± 4.59	5.05 ± 0.46	0.961
Female		4.87 ± 0.36	4.81 ± 0.38	4.72 ± 0.36	0.471
PGA		162.15 ± 112.44	125.81 ± 92.66	114.74 ± 75.23	0.058
PGC		24.82 ± 20.80	17.86 ± 13.95	23.10 ± 20.16	0.182
PGA/PGC		7.91 ± 4.49	7.97 ± 3.70	6.16 ± 3.57	0.06

### Correlations between expression of serum miRNAs and their predicted target PG molecules

**Figure 1:**
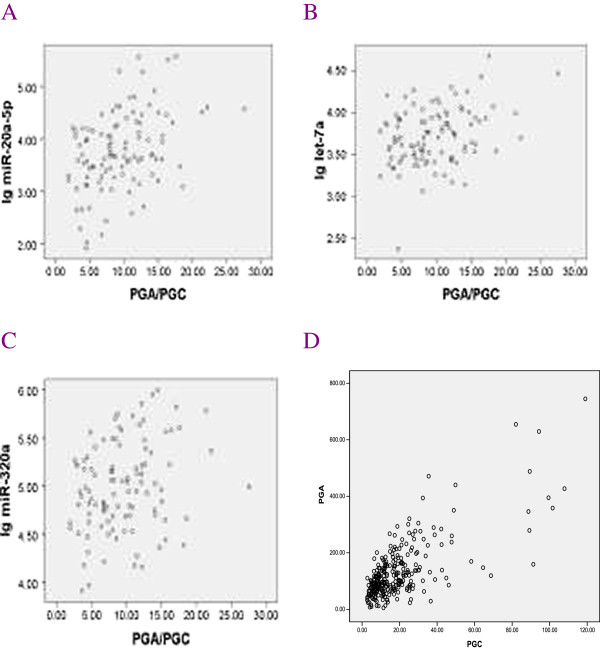
**Scatter plot graph.** Figure 1-**A **- **C**. The scatter plot of serum miRNA levels and their correlations with PGA/PGC ratio from control group. **A**. miR-20a-5p; **B**. let-7a; **C**. miR-320a. Figure 1-**D**. The scatter plot of the correlation between serum PGA and PGC levels. All the scatter plot showed the association analysis of the variants between x and y axis.

For the association analysis, miR-20a-5p expression was not correlated with PGA levels (r = 0.110, *p* = 0.269), and let-7a and miR-320a were not correlated with PGC levels (r = −0.162, -0.102; *p* = 0.102, 0.306, respectively), but they were all positively correlated with the PGA/PGC ratio (r = 0.408, 0.255, 0.324; *p* = <0.001, 0.009, 0.001, respectively) (Figure 1). When compared the studied miRNAs and the predicted target PG molecules with controls, we found serum PGA levels in GC patients were decreased (130.1 ± 107.1 vs. 146.3 ± 101.5, *p* = 0.276), and PGC levels in GC patients were increased (23.1 ± 23.0 vs. 19.2 ± 17.6 *p* = 0.174), though the differences were not significant. The PGA/PGC ratio in the GC group was decreased (7.86 ± 5.46 vs. 9.52 ± 4.85, *p* = 0.039, Table 2), with a correlation coefficient of 0.727 **(**Figure 1). Compared with controls, serum miR-320a levels were decreased (4.90 ± 0.44 vs. 4.99 ± 0.46, *p* = 0.153) and serum PGC levels were increased in the GC group (23.1 ± 23.0 vs. 19.2 ± 17.6 *p* = 0.174), showing apparently opposite tendencies, though the differences were not significant (Table 2).

## Discussion

Recent findings have presented serum/plasma miRNAs as potential biomarkers for several disease conditions, including human cancers [28-31]. However, studies of serum miRNAs in relation to gastric diseases have been limited, and no attention has been paid to miRNAs serially targeting the same gene. Based on a series of miRNAs predicted by bioinformatic software to target to PGA and/or PGC, we selected miR-20a-5p, let-7a and miR-320a for further study. We investigated the potential of miR-20a-5p, let-7a and miR-320a in the diagnosis of AG or GC and the correlation of the three miRNAs with their predicted target molecules PGA, PGC and PGA/PGC ratio. The expression of miR-320a was lower in older female GC patients than in controls, suggesting that miR-320a may prove a valuable marker for diagnosing older women with GC. miR-20a-5p, let-7a and miR-320a were positively correlated with the PGA/PGC ratio, which could indirectly reflect the functional status of the gastric mucosa.

Several studies have identified tumour-specific miRNAs alterations in plasma/serum of cancer patients, and have shown the potential of circulating miRNAs as new non-invasive biomarkers for cancer screening [12,32]. The results of our study indicated no differences in serum miR-20a-5p and let-7a levels among the controls, AG and GC groups, while miR-320a expression fell throughout the controls, AG and GC groups. Serum miR-320a levels were lower in female GC patients than in controls, and this difference was especially marked in older women, aged over 60, suggesting that miR-320a may be a new clinical biomarker for GC diagnosis with population specificity. Studies of miR-320a to date have been limited to its roles in regulating the physiological functions of the blood–brain barrier [33], and in intrahepatic cholangiocarcinoma [34,35], colon cancer [36] and childhood leukaemia [37]. To the best of our knowledge, the current study is the first to report that miR-320a could act as a serum biomarker of GC, especially in older women. Although few studies have investigated its mechanisms, some have reported a relationship between its predicted target PGC and gender, while others reported that the PGA/PGC ratio was related to age, but only in women [38,39]. Software has also predicted that miR-320a targets the estrogen-related receptor gamma (http://www.targetscan.org/cgi-bin/targetscan/mamm_31/targetscan.cgi?species=Human&gid=&mir_c=&mir_nc=&mirg=hsa-miR-320). These lines of evidence all suggest that miR-320a may play an important role in the development of GC in women, and could indirectly reflect the functional state of the gastric mucosa. Its use as a biomarker would be associated with the advantages of non-invasion, low cost and higher stability. However, further studies in larger samples are needed to clarify its mechanisms and verify its use as a biomarker in older women with GC. Of course, the changes of miRNAs maybe not only related with the changes of gastric mucosal function conditions, but also related with some other factors, for examples anemia [40], the stimulus of pathogenic microorganism such as *H.pylor *[41] i, some proteins involved in miRNAs processing pathway like Drosha, Dicer and Argonaute [42,43]. The other factors related should be considered in the further studies in the future.

Serum PGA and PGC levels seem to be related to gastric mucosal glandular and cellular quantities, and also indirectly reflect the secretary function of the gastric corpus and/or gastric antrum. PGA is secreted from the gastric corpus and gastric angle, while PGC is secreted from all the stomach, including the corpus, angle and antrum. A decline in the PGA/PGC ratio reflects the development of atrophic lesions on the gastric mucosa [16], and a low PGA/PGC ratio is related to a high risk of GC [38,44,45]. This has therefore been used as an effective parameter for screening individuals at high risk of GC in the population, and may be a clinically useful biomarker of GC and precancerous diseases such as AG [16,38,46,47]. Broutet et al. reported that although PGC was secreted from different sites in the stomach, it was related to PGA; while PGA increased rapidly, PGC varied slightly, and the correlation coefficient reached 0.75 [38]. The current study also calculated a correlation coefficient between PGA and PGC of 0.727. This relationship may explain why the PGA/PGC ratio reflects the functional status of the gastric mucosa more precisely than either PGA or PGC alone. Our study found no relationship between the expression of miR-20a-5p and PGA, let-7a and PGC, or miR-320a and PGC, but there were positive correlations between each of the three and the PGA/PGC ratio which was already known as a useful biomarker for disease diagnosis, and the studied miRNAs were synchronous with the known PGA/PGC ratio suggesting their potential as useful biomarkers for disease diagnosis.

Considering *H. pylori* is an important environmental factor in the stomach which is also a class Icarcinogen defined by World Health Organization (WHO), we analysis the three miRNAs in the HP stratification among different gastric diseases, but found no difference. Whether *H.pylori* and/or other environmental factors affect this association needs more studies in larger samples in the future.

There was some limitation in our study. First, the selected miRNAs may functionally target PGA/C and/or at least was only proof by the bioinformation software, some evidence for the further functional experiments are needed. Secondly, correlation of expression in the tissue of miRNAs and its predicted target protein is needed for further investigation. Thirdly, the correlation of miRNAs expression and clinicopathological characteristics of patients with gastric cancer, such as Borrmann’s classification, growth pattern, TNM stage, invasion depth and lymph node metastasis,et al. were not further analyzed because of biopsy-limited information missing. Fourth, the source of controls was not the normal population but with a minimal superficial gastritis according to the pathological diagnosis, and gastritis was also belongs a cascade of GC development.

## Conclusion

In conclusion, we demonstrated that serum miR-320a levels were lower in female GC patients aged over 60 than in controls, and may thus represent a valuable marker for diagnosing GC in this group. And the levels of miR-20a-5p, let-7a and miR-320a were positively correlated with PGA/PGC ratio, which could indirectly reflect the functional status of the gastric mucosa.

## Abbreviations

miRNA: microRNA; PG: Pepsinogen; PGA: Pepsinogen A; PGC: Pepsinogen C; qRT-PCR: quantitative reverse transcription polymerase chain reaction; ELISA: Enzyme linked immunosorbent assay; ROC curve: Receiver operating characteristic curve; AUC: The area under curve; nt: nucleotide; cDNA: complementary DNA; AG: Atrophic gastritis; GC: Gastric cancer.

## Competing interests

All authors read and approved the final manuscript, and do not have a commercial or other association that might pose a conflict of interest.

## Authors’ contributions

YY conceived and designed this study and revised the manuscript. QG-D was responsible for the experiment and performed data interpretation. QX involved in writing the paper and revising manuscript critically for important intellectual content. LP-S participated in the design of the study partly. CY-H performed the statistical analysis. All authors read and approved the final manuscript.

## Pre-publication history

The pre-publication history for this paper can be accessed here:

http://www.biomedcentral.com/1472-6890/13/11/prepub
